# Design, Structural Stability, Membrane Binding, and Antibacterial Activity of Novel Antimicrobial Peptides Derived from Wuchuanin-A1

**DOI:** 10.3390/life15101568

**Published:** 2025-10-08

**Authors:** Rizki A. Putri, Ahmad Habibie, Prajnaparamita Dhar, Krzysztof Kuczera, Respati Tri Swasono, Muhammad Saifur Rohman, Tri Joko Raharjo, Teruna J. Siahaan

**Affiliations:** 1 Department of Chemistry, Faculty of Mathematics and Natural Sciences, Universitas Gadjah Mada, Sekip Utara, Yogyakarta 55281, Indonesia; rizkiamaliaputri0198@mail.ugm.ac.id (R.A.P.); ahmad.habibie@mail.ugm.ac.id (A.H.); respati@ugm.ac.id (R.T.S.); 2 Department of Pharmaceutical Chemistry, School of Pharmacy, The University of Kansas, Simons Laboratories, 2095 Constant Ave., Lawrence, KS 66045, USA; 3 Department of Chemical and Petroleum Engineering, School of Engineering, The University of Kansas, Eaton Hall, 1520 W 15th St, Lawrence, KS 66045, USA; prajnadhar@ku.edu; 4 Department of Chemistry, College of Liberal Arts & Sciences, The University of Kansas, Gray-Little Hall, 1567 Irving Hill Rd, Lawrence, KS 66045, USA; kkuczera@ku.edu; 5 Department of Molecular Biosciences, College of Liberal Arts & Sciences, The University of Kansas, Haworth Hall, 1200 Sunnyside Avenue, Lawrence, KS 66045, USA; 6 Department of Agricultural Microbiology, Universitas Gadjah Mada, Jl Flora, Kompleks Bulaksumur, UGM, Yogyakarta 55281, Indonesia; saifur@ugm.ac.id

**Keywords:** α-helical peptides, amphipathic helix, peptide modification, antibacterial, CD spectroscopy, NMR, molecular dynamics simulations, membrane disruption

## Abstract

Antibiotic resistance is a major health problem globally, highlighting the need for alternative antimicrobials that may potentially reduce the emergence of resistance compared to conventional antibiotics. Antimicrobial peptides (AMPs) are promising candidates because of their broad-spectrum activity. In this study, we designed three derivatives (i.e., Analog-1, -2, and -3) of the native peptide, Wuchuanin-A1, for improving their antibacterial activity against *Staphylococcus aureus* and *Escherichia coli*. The hypothesis is that the antibacterial activity of these peptides can be improved by increasing their amphipathicity (evaluated using hydrophobic moment analysis), α-helical stability, and membrane binding properties. In this case, the residues of native peptide were mutated to form an amphipathic peptide, referred to here as Analog-1. Then, the N- and C-termini of Analog-1 were capped with acetyl and amide groups, respectively, to produce Analog-2. Finally, the Asp and Arg residues in Analog-2 were mutated to Glu and Lys residues, respectively, in Analog-3. Circular dichroism (CD) spectra in trifluoroethanol (TFE) or methanol (MeOH) showed that Analog-3 has the highest α-helical stability, followed by Analog-2 and Analog-1. Two-dimensional nuclear magnetic resonance (NMR) spectroscopy and molecular dynamics (MD) simulations studies indicated that Analog-2 and -3 have a stable continuous α-helical structure. Both Analog-2 and -3 can form dimer or oligomer at higher concentrations. All three analogs can bind to model membranes of Gram-positive and Gram-negative bacteria, with Analog-3 as the best membrane binding affinity through Langmuir monolayer analysis. Both Analog-2 and -3 have better antibacterial activities against *S. aureus* and *E. coli* compared to Analog-1 and the native peptide, with minimum inhibitory concentration (MIC) values 3.91 µg/mL against *S. aureus* and 62.5 µg/mL against *E. coli*, which are 2–32-fold lower than those of Analog-1. In addition, Analog-2 and -3 have better activity against *S. aureus* than *E. coli* bacteria. We proposed that the increase in antibacterial activity of Analog-2 and -3 was due to the α-helical stability, amphipathic structure, and membrane binding properties.

## 1. Introduction

Antibiotic resistance is a major global health problem that requires an urgent need for new antimicrobial strategies. The overuse and misuse of antibiotics in human healthcare and agricultural animals have significantly contributed to the emergence of resistant bacterial strains, making conventional treatments less effective and highlighting the need for alternatives [1]. It has been suggested that AMPs can target bacteria with mechanisms to disrupt bacterial membranes that are less prone to the development of drug resistance [2]. However, there are cases that show bacterial resistance to AMPs [2].

AMPs have emerged as a promising alternative to traditional antibiotics due to their unique mechanisms of action. AMPs can directly target and disrupt bacterial cell membranes, leading to cell lysis and bacterial death [3]. This membrane-targeting mechanism of AMPs makes them less likely to generate drug resistance compared to traditional antibiotics, which typically target bacterial proteins or nucleic acids [4]. Among AMPs, α-helical peptides have potential to be effective antibiotics. Notable examples for AMPs include magainin-2, which has an α-helical structure and was originally isolated from the skin of the African clawed frog (*Xenopus laevis*) [5,6], and pexiganan (MSI-78) that is derived from the magainin family [7,8]. Pexiganan is a synthetic peptide that has advanced to phase III clinical trials [7,8]. Magainin-2 is an effective antimicrobial able to disrupt bacterial membranes due to its α-helical structure and amphipathic nature [9,10]. A combination of amphipathicity and α-helical structure enables AMPs to create membrane pores or disturb membrane integrity, leading to bacterial cell leakiness and lysis [11]. These features of AMPs contribute to their (a) broad-spectrum activity as antibacterials, (b) reduced potential of developing resistance, and (c) enhanced membrane-disrupting capabilities [12].

Wuchuanin-A1 is a naturally occurring peptide derived from the skin of frogs called *Odorrana wuchuanensis*, with antimicrobial, antioxidant, and antifungal activities [13]. However, the native form of Wuchuanin-A1 lacks the necessary structural features to be an optimal antibacterial. Inspired by the success of magainin-2 and its derivatives, we hypothesize that the antibacterial activity of Wuchuanin-A1′s analogs can be increased by enhancing their α-helical stability and amphipathicity through structural modifications. It is expected that the increase in α-helical stability can improve the bacterial membrane binding and the activity against target Gram-positive and Gram-negative bacteria [14].

The primary aim of this study is to enhance the α-helical content, amphipathicity, and antibacterial activity of Wuchuanin-A1 analogs by rational peptide design. In this study, three different analogs (Analog-1, -2, and -3) of Wuchuanin-A1 were designed by mutations of all helix breaker residues such as Pro and Gly residues. Then, the amphipathicity of the structure was optimized and verified by calculating the hydrophobic moment, replacing hydrophilic residues at the helix hydrophobic face of the native peptide with helix former hydrophobic residues. Conversely, the hydrophobic residues in the helix hydrophilic face were replaced with helix former residues with hydrophilic property. Finally, N- and C-termini of the peptide were capped with acetyl and amide groups, respectively. The α-helix conformation of the native peptide and its analogs were evaluated with CD spectroscopy. The solution structures of Analog-2 and -3 were determined using a combination of 2D-NMR spectroscopy and MD simulations. The membrane binding properties of these peptides to models of Gram-positive and Gram-negative membranes were determined by tensiometry, using a Langmuir trough. The antibacterial activity of each peptide was assessed in inhibiting bacterial growth of *E. coli* and *S. aureus* bacteria.

## 2. Materials and Methods

### 2.1. Peptide Preparation

Synthetic peptides with a purity greater than 95% were obtained from DgPeptides (Hangzhou, China). Each peptide was analyzed using analytical HPLC and Mass Spectrometry (Table 1, Supplementary Figure S1). Stock solutions of each peptide were prepared by dissolving them in 10 µM potassium phosphate buffer pH 7.

### 2.2. Peptide Analysis by CD Spectroscopy

CD spectra were recorded using a Jasco J-815 Spectropolarimeter equipped with a Peltier temperature controller (Jasco PTC-424S) in 1.0 mm quartz cells. Measurements were taken from 190 to 250 nm with a 0.5 nm step size, 50 nm/min scan speed, and 1.0 nm bandwidth. Each sample was scanned five times. Background correction was performed by subtracting the contribution of the buffer spectrum. A peptide concentration of 200 µg/mL was prepared in solutions containing varying concentrations of trifluoroethanol (TFE) and methanol (MeOH). Peptide stability was assessed under different temperatures (10 to 90 °C) and pHs. Potassium phosphate and citrate buffers were used for pH 3.0, 4.0, 5.0, and 6.0; potassium phosphate buffer was used for pH 8.0. CD measurements for pH-dependent studies were conducted in triplicate. CD spectra were plotted as mean residue molar ellipticity (MRE, degree × cm^2^ × dmol^−1^) versus wavelength (λ, nm). All the mean helicity values (*f_H_*) were calculated by using Equation (1) [15]. Additionally, peptide dimerization or oligomerization was determined in 20% TFE using various concentrations of Analog-1, -2, and -3.

(1) $$f_{H}=\frac{{\left[\theta \right]}_{222}-(1550-40T)}{\left(-42,400+140T\right)\left(1-\frac{4.8}{N_{pep}}\right)-\;(1550-40T)}$$

### 2.3. Effect of Concentrations on Peptide Oligomerization by CD Spectroscopy

To evaluate peptide dimerization or oligomerization, CD spectra were obtained for peptide solution at various concentrations. The *θ* at 222 nm was plotted as a function of peptide concentrations to monitor changes in α-helicity associated with oligomerization. The Honda method was used to analyze peptide oligomerization by fitting *θ* data to a two-state model, describing the equilibrium between monomeric and oligomer forms [16]. The Honda equation (Equation (2)) was used to estimate the dissociation constant (*K_d_*) for oligomerization, where *θ* represents the observed molar ellipticity at a given total peptide concentration (*C_t_*), *θ_m_* is the molar ellipticity of the monomeric form, and *θ_d_* is the ellipticity of the oligomeric form. This analysis provided insight into the tendency of peptide to form dimer or oligomers at different concentrations [16].

(2) $$\theta =\theta_{d}+(\theta_{m}-\theta_{d})\frac{-K_{d}+\sqrt{K_{d}^{2}+{4C}_{t}K_{d}}}{2C_{t}}$$

### 2.4. Nuclear Magnetic Resonance (NMR) Analysis

The solution conformation of Analog-2 and -3 (Table 1) were determined using 2D-NMR. Both peptides were dissolved in 20% deuterated methanol (MeOD) at a concentration of 1–2 mM. NMR spectra were recorded at 298.15 K using a Bruker Avance III 600 MHz NMR spectrometer. One-dimensional ^1^H NMR (1D-NMR) spectra were collected to provide initial chemical shift information and coupling constants of the NH (^3^*J_NH-HCα_*) for each amino acid. Two-dimensional NMR experiments, including Correlation Spectroscopy (COSY), Total Correlation Spectroscopy (TOCSY), and Nuclear Overhauser Effect Spectroscopy (NOESY) were performed to elucidate structural details of these peptides [17]. COSY spectrum was employed to identify through three-bond interaction between protons, while TOCSY spectrum was used to determine proton spin systems within each amino acid. NOESY spectrum was utilized to detect through-space proton interactions that were used to determine peptide sequential assignment, as well as its conformation in solution. MNOVA software was used to process NMR spectra for visualization and integration proton–proton interactions.

(3) $$r_{x}=r_{std}\sqrt[{6}]{\left(\frac{I_{std}}{I_{x}}\right)}$$

Interproton distances within the peptide were obtained using NOESY spectra. These distances (*r_x_*) were calculated by comparing the NOE cross-peak intensities with a known distance reference. The distance between geminal protons, set at 1.77 Å, was used as the reference point (*r_std_*). The calculation using Equation (3) followed the relation, where *r_x_* is the distance between protons, *r_std_* is 1.77 Å, *I_std_* represents the NOE intensity of geminal protons, and *I_x_* is the NOE intensity between the interacting protons [17,18].

The ^3^*J_NH-HCα_* coupling constant (^3^*J_HNα_*) was extracted from the backbone amide region in the ^1^H spectra at 298.15 K, given in Hertz. To predict the relationship between this coupling constant and the dihedral angles, a parameterized form of the Karplus equation (Equation (4)) was used, where the angle φ is defined as *θ* = ∣φ − 60°∣ [17,18,19].

(4) $${3J}_{HN\alpha }=6.4\;{cos}^{2}\theta -1.4\;cos\;\theta +1.9$$

### 2.5. Molecular Dynamics (MD) Simulations

The 3D structure of the peptide backbone was calculated using Ambiguous Restraints for Iterative Assignment (ARIA), incorporating dihedral and distance restraints obtained from NOE spectra. After an initial structure calculation, a refinement process generated 10 candidate structures, with the final model selected based on PROCHECK analysis for minimal structural violations and optimal stereochemistry [20,21].

The peptide structure was then modified using the CHARMM-GUI solution builder [22,23]. Acetyl and amide groups were added to the N- and C-termini, respectively. The peptides were solvated in a cubic box with TIP3P water molecules [24], and a Monte Carlo ion replacement method with KCl was used to neutralize the system. Simulations were conducted with GROMACS 5.0.4 [25] using the CHARMM36 force field [26]. Energy minimization was carried out to eliminate steric clashes and unfavorable contacts before simulations proceeded.

Following system minimization, a 10 ns NPT equilibration was carried out at 298.15 K and 1 bar. For the NMR-constrained MD simulation, distance and dihedral angle restraints from NOE data were used with a time step of 2 fs and a total production run of 100 ns [17]. Temperature was maintained at 298.15 K using the velocity-rescale thermostat [27], and non-bonded interactions were managed by the Particle Mesh Ewald (PME) method [28], with a 1.2 nm cutoff for both Coulombic and van der Waals interactions. Hydrogen-containing bonds were constrained using the LINCS algorithm [29], permitting the larger time step of 2 fs. Root-mean square deviation (RMSD) calculations were performed using the backbone atoms (N, Cα, and C) of each residue to assess structural stability. Initial coordinates from the production step served as the reference structure. The 10 lowest-energy structures were selected for further analysis and visualized with Discovery Studio and PyMOL [30].

### 2.6. Membrane–Peptide Interaction Analysis

In this study, the Langmuir monolayer technique was employed to investigate the interactions between modified peptides and model membranes. The phospholipids used were 1-palmitoyl-2-oleoyl-sn-glycero-3-phosphoethanolamine (POPE) and 1-palmitoyl-2-oleoyl-sn-glycero-3-phosphoglycerol (POPG) (Avanti, Alabaster, AL, USA). Gram-positive and Gram-negative model membranes were constructed with different phospholipid ratios. Specifically, a 7:3 ratio of POPG to POPE was used to model Gram-positive bacterial membranes, whereas a 3:7 ratio of POPG to POPE was used to represent Gram-negative bacterial membranes.

In the Langmuir trough method, a lipid monolayer was formed by spreading the POPE:POPG prepared in chloroform–methanol mixture, dropwise onto the surface of a potassium phosphate buffer at pH 7.0 to form a precise single-molecule-thick arrangement of lipids at the air–buffer interface. Peptides were subsequently injected into the buffer solution below the lipid monolayer through a hole at the bottom part of the lipid monolayer, and their adsorption onto the lipid surface was monitored using a Wilhelmy plate that measures the surface pressure of the monolayer. Changes in the surface pressure of the lipid monolayer provide quantitative insights into the peptide–membrane interactions.

Surface pressure, denoted as *π*, is a crucial parameter in this context. It is defined as the difference between the surface tension of the pure subphase (*γ*_0_) and the surface tension of the subphase with the lipid monolayer (*γ*), as expressed in (Equation (5)) [31].

(5) $$\pi =\gamma_{0}-\gamma$$

To determine the effect of peptide adsorption on the lipid monolayer, the surface pressure was measured before and after peptide addition. Following the injection of peptides, the surface pressure was measured again. The difference in these measurements (Δ*π*) indicates the extent of peptide adsorption into the membrane. An increase in surface pressure (Δ*π* > 0) after the addition of peptides suggests that the peptides have successfully adsorbed onto the lipid monolayer, thereby increasing the packing density and altering the intermolecular interactions within the membrane.

### 2.7. Antibacterial Activity of Peptides

The antibacterial activity of peptide was tested against Gram-positive *S. aureus* (ATCC 25923) and Gram-negative *E. coli* (ATCC 25922) bacteria using the broth microdilution method. Chloramphenicol, a broad-spectrum antibiotic, was used as a positive control. First, the bacteria were cultured in MHB media, then incubated overnight at 37 °C in a shaker incubator. Then, MHB media was added to the 96-well microplates; the peptide previously dissolved in PBS buffer at pH 7.4 was added into the 96-well. The microdilution series used was a two-fold series. Finally, the overnight culture bacteria that has been diluted in MHB media (10^5^–10^6^ CFU/mL) was added to the well. The final volume of the mixture for absorbance measurement was 100 µL. The final concentration of bacteria used was ~5 × (10^4^–10^5^) CFU/mL, according to the method recommended by the Clinical lab standards institute (CLSI) VET03 [32]. The test was performed in triplicates. Bacteria in PBS with MHB media without any peptide was used as a positive control to monitor the normal growth of bacteria and MHB media only as a negative control. Then, the mixture was incubated at 37 °C overnight, and its absorbance was measured using a microwell reader. The MIC values were defined as the lowest concentration showing no visible growth after overnight incubation at 37 °C [33].

## 3. Results

### 3.1. CD Spectroscopy to Determine Secondary Structures of Peptides

The effects of helical stabilizer (i.e., TFE and MeOH) and environmental factor (i.e., temperature and pH) on the peptide conformation were evaluated for the native peptide Wuchuanin-1 and its analogs (Analog-1, -2, and -3) using CD spectroscopy. The secondary structure of Wuchuanain-1 and its derivatives under these various conditions were determined against their helical contents. CD spectroscopy studies were carried out at a concentration of 200 µg/mL.

#### 3.1.1. The Effect of TFE on α-Helical Conformation

**Figure 1:**
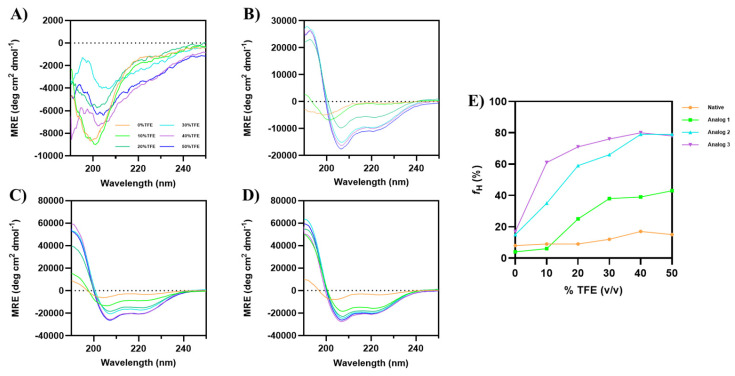
CD spectra of Wuchuanin-1 and its analogs in various concentrations of TFE: (**A**) Native peptide, (**B**) Analog-1, (**C**) Analog-2, and (**D**) Analog-3. The peptide concentration was 200 µg/mL. Analog-1 showed the helical structure starting from 20% TFE, while Analog-2 and -3 exhibited the highest α-helicity even at lower TFE concentrations (<20%). (**E**) The changes in percentage of α-helix fraction (*f_H_*) as a function TFE concentrations were calculated using Equation (1). The *f_H_*s of Analog-2 and -3 were similar at 40–50% TFE.

The analysis was performed in varying concentrations of TFE, ranging from 0% to 50% TFE. The addition of TFE promoted α-helical formation for all the analogs, while minimal α-helical change was observed for the native peptide (Figure 1). The formation of α-helix conformation was clearly observed in the buildup of absorption minima at 208 nm and 222 nm, along with the positive signal at 193 nm [34].

Although there were changes in the CD spectra of the native peptide (Figure 1A) upon addition of TFE, no α-helix formation was observed independently from TFE concentration. In contrast, Analog-1 started to form an α-helical structure at 20% TFE, with a maximum change in helicity signals at 50% TFE (Figure 1B). Analog-2 exhibited high helicity without TFE, with optimal signals at 40–50% TFE (Figure 1C). Analog-3 also formed an α-helix without TFE, with optimal helicity at 40–50% TFE, and it has higher helicity than Analog-2 at 10–30% TFE (Figure 1D). The percentage of α-helical content (*f_H_*) was calculated using Equation (1), highlighting the structural differences among the peptides (Figure 1E) [35]. Analog-3 has a maximum helical content of 78–80% in 40–50% TFE. Similarly, Analog-2 has a maximum helical content of 79% in 40–50% TFE. Analog-3 has higher helicity (61%) than Analog-2 (35%) at 10% TFE (Figure 1E). Analog-1 reached a maximum helical content of 39–43% in 40–50% TFE, while the native peptide has 15–17% helical content in 40–50% TFE. Overall, TFE enhanced the α-helical structure to promote intramolecular hydrogen bonding and reduce interactions between the peptide and water molecules. This effect is consistent with a previous study by Rivera-Najera et al. (2014) in which TFE can induce and stabilize α-helix structure by mimicking membrane-like conditions [36].

#### 3.1.2. The Effect of MeOH on α-Helical Conformation

**Figure 2:**
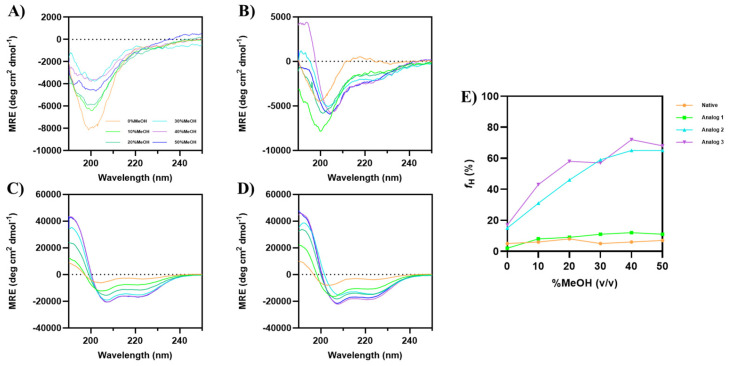
CD spectra of Wuchuanin-1 and its analogs in the various concentrations of MeOH: (**A**) Native peptide, (**B**) Analog-1, (**C**) Analog-2, and (**D**) Analog-3. The peptide concentration was 200 µg/mL. (**A**) The native peptide showed spectral changes with increasing MeOH concentrations, but there was no enhancement in α-helical signals at 208 and 222 nm. (**B**) Increasing concentration of MeOH weakly induced the formation of α-helical signals in Analog-1. Both (**C**) Analog-2 and (**D**) Analog-3 have α-helical signals in the absence of MeOH. The intensity of minima at 208 and 222 nm enhanced for both Analog-2 and -3 as the MeOH concentration increased. (**E**) The changes in percentage of α-helix fraction as a function of MeOH concentrations were calculated using the mean helicity value (*f*_H_) equation. The *f_H_*s of Analog-3 were higher than Analog-2 as the MeOH concentrations increased and the *f_H_*s maximized in 40–50% MeOH for Analog-2 and -3.

**Figure 3:**
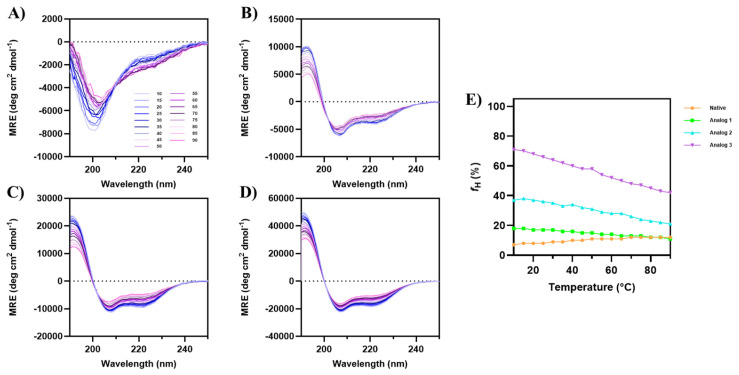
The effect of temperature from 10 °C to 90 °C on the CD spectra of (**A**) native peptide, (**B**) Analog-1, (**C**) Analog-2, and (**D**) Analog-3 in 20% TFE. (**E**) The fraction of helical content (*f_H_*) plotted against temperature for all peptides. Initially at 10 °C, the native peptide has very low α-helical signal; however, the helical signals at 208 and 222 nm increased when temperature was increased to 90 °C. All three analogs maintained consistent α-helical features, with negative peaks at 208 nm and 222 nm, and a positive peak at 193 nm, across all temperatures. The depth of the signals at 208 nm and 222 nm for all analogs were decreased as the temperature increased to 90 °C.

Increasing concentrations of MeOH was also used to induce the formation of α-helical structure of each peptide (Figure 2). Similarly to the previous result with TFE, MeOH did not induce the α-helix signals at 208 and 222 nm in the native peptide, even up to 50% MeOH (Figure 2A); in contrast, Analog-1 showed a small increase in α-helix signals upon increasing MeOH concentrations. Analog-2 (Figure 2C) and -3 (Figure 2D) displayed a well-defined helix even in the absence of methanol, and the addition of MeOH further enhanced the α-helical signals with a plateau at 40–50% MeOH. The helical content (*f_H_*) for the native peptide did not change at all different MeOH concentrations (Figure 2E). In contrast, Analog-1 enhanced *f_H_* from 2% to 9% in 0 to 20% MeOH, followed by a plateau at *f_H_* of 11–12% in 30–50% MeOH. The helical contents Analog-2 and -3 were maximized at *f_H_* of 65% and 72%, respectively, in 40–50% MeOH.

#### 3.1.3. The Effect of Temperature on α-Helical Conformation

The effect of temperature on the helical stability of each peptide in 20% TFE was determined from 10 °C to 90 °C (Figure 3), and the data showed that Analogs-1, -2, and -3 maintain relatively stable α-helical structure. For the native peptide, the increase in temperature decreases the minima at 200 nm, while the minima at 208 and 222 nm increase (Figure 3A). In contrast, the intensities of minima at 222 and 208 nm for all three analogs were reduced at the increase in temperature (Figure 3B–D), indicating that the helix stability was reduced as the temperature increased. For Analog-3, the initial fraction of helicity (*f_H_*) at 10 °C was 71%, while the *f_H_* at 90 °C was 42%; therefore, the change in fraction of helicity vs. temperature (Δ*f_H_*/ΔT) is 0.363% per degree. The initial *f_H_*s for Analog-2 and -1 at 10 °C were at 37% and 18%, respectively; the final *f_H_*s of Analog-2 and -1 at 90 °C were 21% and 11%, respectively (Figure 3E). Therefore, the Δ*f_H_*/ΔT values for Analog-2 and -1 were 0.2% per degree and 0.087% per degree. Thus, the drop of helical contents against temperatures (Δ*f_H_*/ΔT) occurred in the following order Analog-3 > -2 > -1. It was interesting to note in native peptide that the increase in temperatures from 10 °C-to-90 °C increased the *f_H_* from 7% to 12%. Overall, during the increase in temperature in all three analogs, the transition from the folded to unfolded state occurred gradually, without the fully unfolded structure at the highest temperature (90 °C). Because the α-helical structure did not completely unfold at 90 °C, a precise melting temperature (Tm) cannot be determined. These observations suggest that all analogs exhibit remarkable thermal stability, maintaining partial helical structure even at temperatures up to 90 °C.

#### 3.1.4. The Effect of pH on α-Helical Conformation

**Figure 4:**
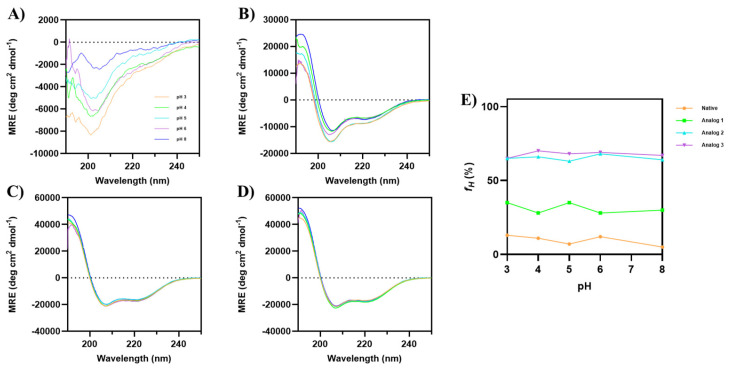
The effects of pH 3–8 on the CD spectra of (**A**) native peptide, (**B**) Analog-1, (**C**) Analog-2, and (**D**) Analog-3 in 20% TFE. All analogs maintain their helical conformation despite pH changes. The CD spectra of Analog-1 at pH 3–8 show slight fluctuations, indicating minor changes in helical stability. (**E**) The fraction of helical structure (*f_H_*) was plotted against pH, and it showed that both Analog-2 and -3 have helical stability at pH 3–8.

The helical stability of all peptides was examined as a function of pHs (3–8) in 20% TFE (Figure 4). Although the parent native peptide did not have a helical structure, pH changes showed a dramatic decrease in the minima intensity at 200 nm (Figure 4A), with minimal changes in the α-helical signals at 208 and 222 nm. Analog-1 showed a slight change in the CD spectra as the pH increased, with some signal fluctuations at pH 3 and 5 (Figure 4B). In contrast, the CD spectra of Analog-2 and -3 did not show any change throughout all pHs (Figure 4C,D), indicating that the α-helical structure was stable at various pHs. Analog-1, *f_H_* values fluctuated between 28 and 35% at all pHs (Figure 4E). The native peptide has an *f_H_* value of 13% at pH 3, with lower *f_H_* at pH 5 (7%) and 8 (5%). In contrast, both Analog-2 and -3 have a stable helical structure in all pHs (Figure 4E). Overall, substituting specific amino acids in the native peptide with helix-promoting residues resulted in a high α-helical content with good stability at different temperatures and pH values.

### 3.2. Oligomerization Properties of Analogs

The potential oligomerization of these analogs was determined by observing the CD spectra at 222 nm as a function of concentration. A non-linear change in the spectra at 222 nm as a function of concentration indicates a potential formation of dimer or oligomers. The Honda equation was utilized to calculate the *K_d_* of oligomerization of peptides by fitting the changes in CD molar ellipticities as a function of peptide concentrations [16]. At higher concentrations, Analog-3 formed stronger dimer or oligomer with *K_d_* = 2.5 × 10^−5^ M compared to Analog-2 with *K_d_* = 1 × 10^−4^ M and Analog-1 with *K_d_* = 2 × 10^−4^ M (Table 2). The results suggest that the capping of the N- and C-termini enhanced the helical conformation but did not promote dimer or oligomer formation. Substituting aspartic acid with glutamic acid and arginine with lysine further enhanced the helical stability and oligomeric formation of Analog-3 compared to Analog-2.

### 3.3. Conformational Study of Analog-2 by NMR and MD Simulations

#### 3.3.1. COSY, TOCSY, and NOESY Spectra

**Figure 5:**
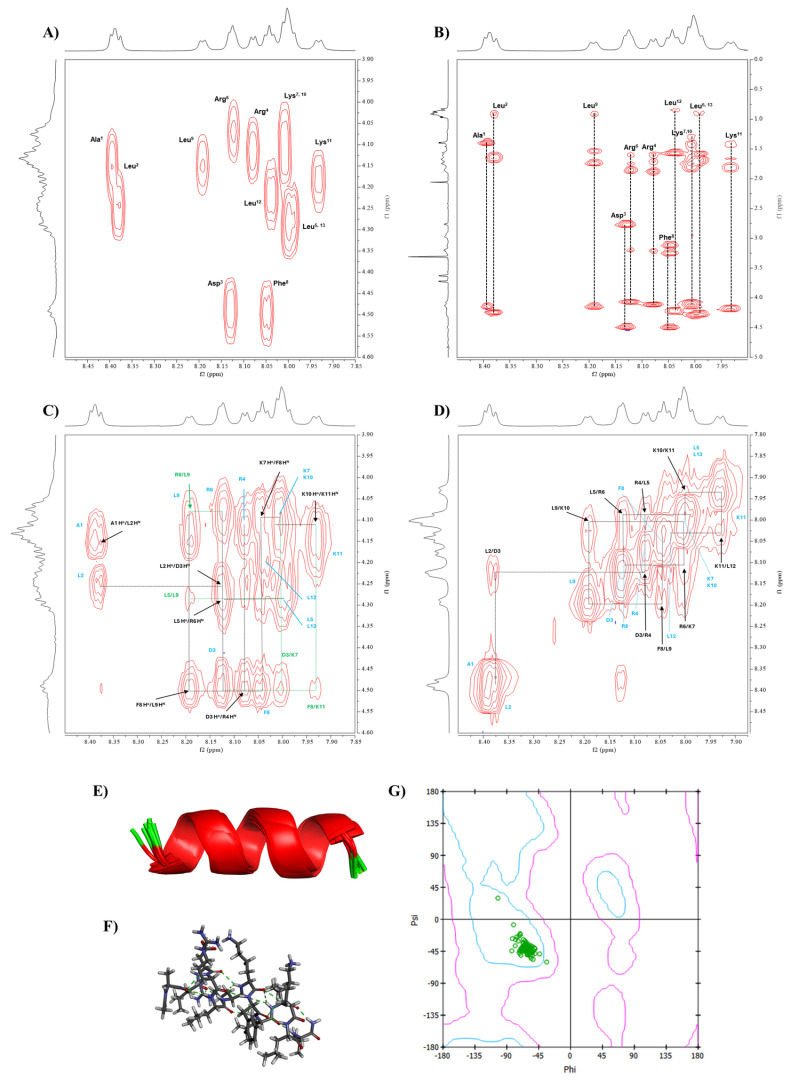
The conformational study of Analog-2 using NMR and molecular dynamic (MD) simulations. The NMR spectra of Analog-2 were performed in 20% MeOD at 298.15 K using a Bruker AVIII 600 MHz spectrometer. (**A**) COSY and (**B**) TOCSY spectra were used to assign all protons in Analog-2. The through-space proton–proton interactions were determined using NOESY spectra using (**C**) Hα_(*i*)_-NH_(*i*+1)_ and (**D**) NH_(*i*)_-NH_(*i*+1)_ interactions. The NMR-restrained MD simulations generated α-helical structure, as represented by (**E**) ribbon and (**F**) stick structures. (**G**) The Ramachandran plot was constructed to show the Phi-Psi dihedral angles of Analog-2 that are located in the α-helical region.

Because Analog-2 has a stable α-helical conformation, its solution conformation was determined using MD simulations with NOE-distance and Phi angle constraints from NMR data [17]. To accomplish this goal, proton assignments were carried out using 2D-COSY (Figure 5A) and 2D-TOCSY (Figure 5B) NMR spectra [17]. COSY spectrum identified proton–proton connectivity through three bonds, such as the NH-to-Hα connectivity (Figure 5A). All NH-to-Hα connectivities within the peptide were identified in the COSY spectrum. In the region between 7.98 and 8.01 ppm, there were overlapping NH signals from four different amino acids such as from two lysines at 8.00 and 8.01 ppm, as well as two leucines at 7.98 and 7.99 ppm. A complete proton assignment for each amino acid spin system was accomplished using the characteristic pattern for each amino acid in TOCSY spectra (Figure 5B). A total of 11 distinct spin systems were identified for Analog-2. Despite spectral overlap, the differences between these amino acids were distinguished using sequential correlations in the NOESY spectra, which helped to determine the position of each amino acid within the peptide. Finally, the chemical shifts in all identified protons are shown in the Supplementary Table S1.

The NOESY spectra confirmed the sequential connectivity of amino acids within the peptide to complement proton identification for the COSY and TOCSY spectra [17]. The NOESY spectra identified the correlations between Hα(i)-NH(i+1), allowing us to confirm the amino acid sequence in Analog-2 (Figure 5C). In this case, seven cross-peaks were observed for Analog-2 to indicate correlations between Hα_(A1)_-NH_(L2_), Hα_(L2)_-NH_(D3)_, Hα_(D3)_-NH_(R4)_, Hα_(L5)_-NH_(R6)_, Hα_(K7)_-NH_(F8)_, Hα_(F8)_-NH_(L9)_, and Hα_(K10)_-NH_(K11)_ (Figure 5C). The NH_(i)_-NH_(i+1)_ correlations were also assigned to nine cross-peaks, including NH_(L2)_-NH_(D3)_, NH_(D3)_-NH_(R4)_, NH_(R4)_-NH_(L5)_, NH_(L5)_-NH_(R6)_, NH_(R6)_-NH_(K7)_, NH_(F8)_-NH_(L9)_, NH_(L9)_-NH_(K10)_, NH_(K10)_-NH_(K11)_, and NH_(K11)_-NH_(L12)_ (Figure 5D). This result confirmed the presence of a continuous α-helix backbone structure in Analog-2. Medium through-space interactions were also identified, such as NH_(L2)_-NH_(R4)_, NH_(R6)_-NH_(F8)_, and NH_(L9)_-NH_(K11)_. Several additional long-range interactions were also identified between Hα_(i)_-NH_(i+3)_, Hα_(i)_-NH_(i+4)_, and Hα_(i)_-Hβ_(i+3)_.

#### 3.3.2. Phi Dihedral Angles from ^3^J_NH-Hα_ Coupling Constants

Additionally, the Phi dihedral angles on the peptide were determined using the ^3^*J_NH-Hα_* coupling constants from the NMR spectrum using the Karplus equation [17,18]. The measured coupling constants were between 4.8 and 7.2 Hz, and the calculated Phi angles are shown in Table 3. The observed Phi dihedral angle for each amino acid was used as constraints for the MD simulations.

#### 3.3.3. Conformation of Analog-2 from MD Simulations

Interproton distances within Analog-2 were calculated from the NOE cross-peak intensities using Equation (3); the cross-peak intensity of geminal protons was used as a reference distance of 1.77 Å. The calculated interproton distances are listed in Supplementary Table S2 and they were used as constraints in MD simulations to determine the conformation of Analog-2. The result found 10 lowest-energy structures of Analog-2 that have consistency with interproton distance and Phi angle input. The MD simulations showed that Analog-2 has a stable helical structure represented as ribbon (Figure 5E) and stick (Figure 5F) structures. Ramachandran plot was generated to confirm the α-helical structure with most of the Phi and Psi angles from all 10 structures landed in the α-helical region, except for the Leu12 (Figure 5G). The Phi angles from the MD simulations and NMR data were compared in Table 3. The Phi/Psi angles of Analog-2 from MD simulation are listed in the Supplementary Table S3. Overall, Analog-2 has a continuous α-helical structure.

### 3.4. Conformational Study of Analog-3 by NMR and MD Simulations

#### 3.4.1. COSY, TOCSY, and NOESY Spectra

**Figure 6:**
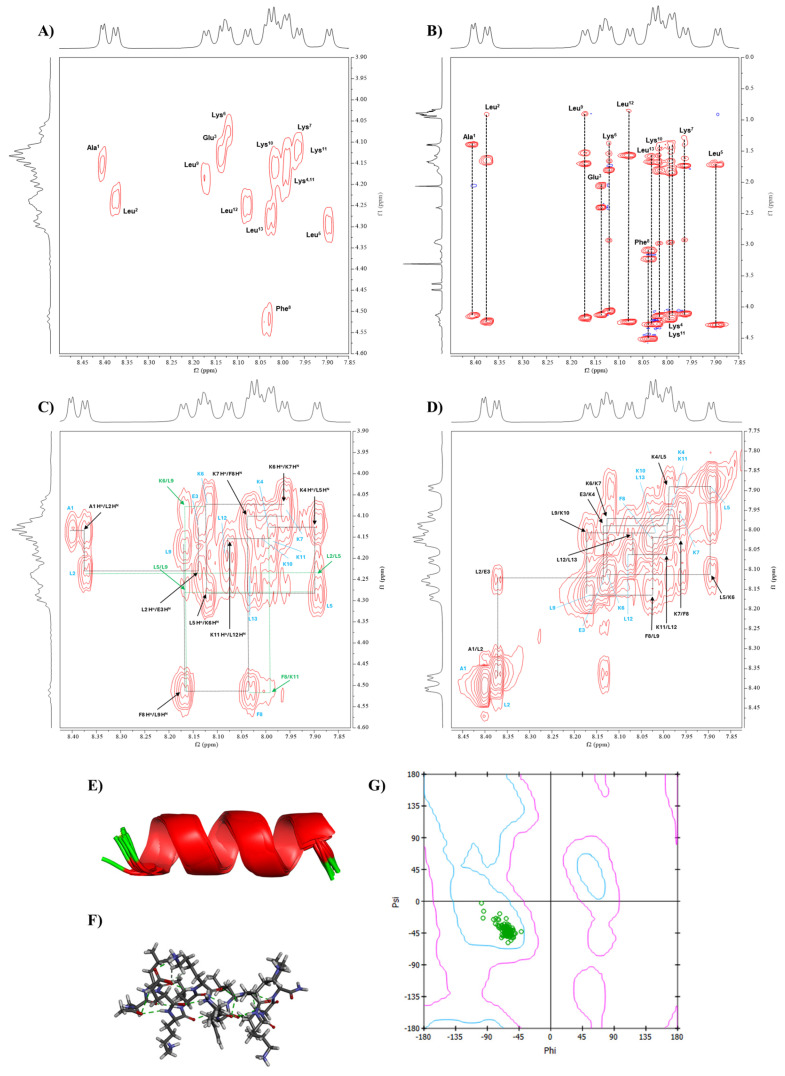
The conformational determination of Analog-3 using NMR and MD simulations. The NMR spectra of Analog-3 were performed in 20% MeOD at 298.15 K using a Bruker AVIII 600 MHz spectrometer. (**A**) COSY and (**B**) TOCSY spectra of were used to assign all protons in Analog-3 peptide. NOESY spectra were used to determine through-space proton–proton interactions between (**C**) Hα_(*i*)_-NH_(*i*+1)_ and (**D**) NH_(*i*)_-NH_(*i*+1)_ protons. The NMR-restrained MD simulations generated α-helical structure, as represented by (**E**) ribbon and (**F**) stick structures. (**G**) Ramachandran plot was generated to show the Phi-Psi dihedral angles of Analog-3 that are located in the α-helical region.

Conformational studies of Analog-3 were also carried out by NMR (Figure 6) and all protons within the peptide were assigned using COSY (Figure 6A) and TOCSY (Figure 6B) spectra [17]. COSY spectra revealed 13 cross-peaks between NH and Hα, with some overlapping cross-peaks from two Lys residues. TOCSY spectra identified a distinct spin system for each amino acid, including the two overlapping NHs of Lys residues at 7.99 ppm. The appearance of two different chemical shifts at 4.11 and 4.2 ppm for their Hα protons was helpful to distinguish the assignment of protons in two overlapping lysine residues. A combination of COSY and TOCSY spectra provided a detail mapping of the spin systems for each amino acid in Analog-3. The full proton assignments of in Analog-3 are shown in Supplementary Table S4.

Similarly to Analog-2, the NOESY spectra of Analog-3 were used to determine the sequential connectivity of the amino acids and validate the assignments made in the COSY and TOCSY spectra. By using the Hα_(i)_-NH_(i+1)_ correlations, the position of each amino in the sequence was determined (Figure 6C). Additionally, NH_(i)_-NH_(i+1)_ cross-peaks were used to confirm the sequential assignment, as well as to indicate the presence of a continuous α-helical structure in Analog-3 (Figure 6D). These cross-peaks include interactions between NH_(A1)_-NH_(L2)_, NH_(L2)_-NH_(E3)_, NH_(E3)_-NH_(K4)_, NH_(K4)_-NH_(L5)_, NH_(L5)_-NH_(K6)_, NH_(K6)_-NH_(K7)_, NH_(K7)_-NH_(F8)_, NH_(F8)_-NH_(L9)_, NH_(L9)_-NH_(K10)_, NH(_K11)_-NH_(L12)_, and NH_(L12)_-NH_(L13)_.

Medium-range interactions were observed through NH_(i)_-NH_(i+2)_ connectivities between NH_(A1)_-NH_(E3)_, NH_(L2)_-NH_(K4)_, and NH_(L5)_-NH_(K7)_. Additional medium-range interactions of Hα_(i)_-NH_(i+3)_ were also found between Hα_(L2)_-NH(_L5)_, Hα_(K6)_-NH_(L9)_, and Hα_(F8)_-NH_(K11)_. Long-range interactions were identified between Hα_(i)_-NH_(i+4)_ that were reflected in cross-peaks between Hα_(L5)_-NH_(L9)_, Hα_(F8)_-NH_(L12)_, and Hα_(L9)_-NH_(L13)_. Long-range interactions between several Hα_(i)_-Hβ_(i+3)_ were found between Hα_(L5)_-Hβ_(F8)_, Hα_(K6)_-Hβ_(L9)_, and Hα_(F8)_-Hβ_(K11)_. All of these short-, medium-, and long-range interactions support the presence of a stable α-helical structure in Analog-3 in solution.

#### 3.4.2. Phi Dihedral Angle from *^3^J_NH-HCα_* Coupling Constant

^3^*J_NH-Hα_* coupling constants of Analog-3 from the NMR spectra were used to calculate the Phi dihedral angles for each amino acid (Table 3). The observed coupling constants were between 4.2 and 6.6 Hz, with the calculated Phi angles between −60 and −78.9 degrees (Table 3). These Phi angles were used as additional constraints in the MD simulations.

#### 3.4.3. Conformation of Analog-3 from MD Simulations

The NOE cross-peak intensities were converted to interproton distances of Analog-3 using Equation (3), with a distance reference of 1.77 Å for geminal protons (Supplementary Table S5). The resulting interproton distances were used in NMR restraints in the MD simulation for Analog-3. The MD simulations produced 10 lowest-energy structures that have consistency with interproton distances (Supplementary Table S5) and Phi angles (Table 3) from the NMR data. A model for the solution structure of Analog-3 has a continuous α-helical backbone structure, as represented in the ribbon (Figure 6E) or stick (Figure 6F) model. To confirm the helical nature of the backbone, the Phi/Psi angles were presented in Ramachandran plot, and the Phi/Psi angles of all the amino acids resided in the α-helical region (Figure 6G). The Phi/Psi angles from the model and NMR data are listed in the Supplementary Table S6. In summary, Analog-3 has a stable and continuous α-helical structure.

### 3.5. Binding of Peptides to Model Membranes

**Figure 7:**
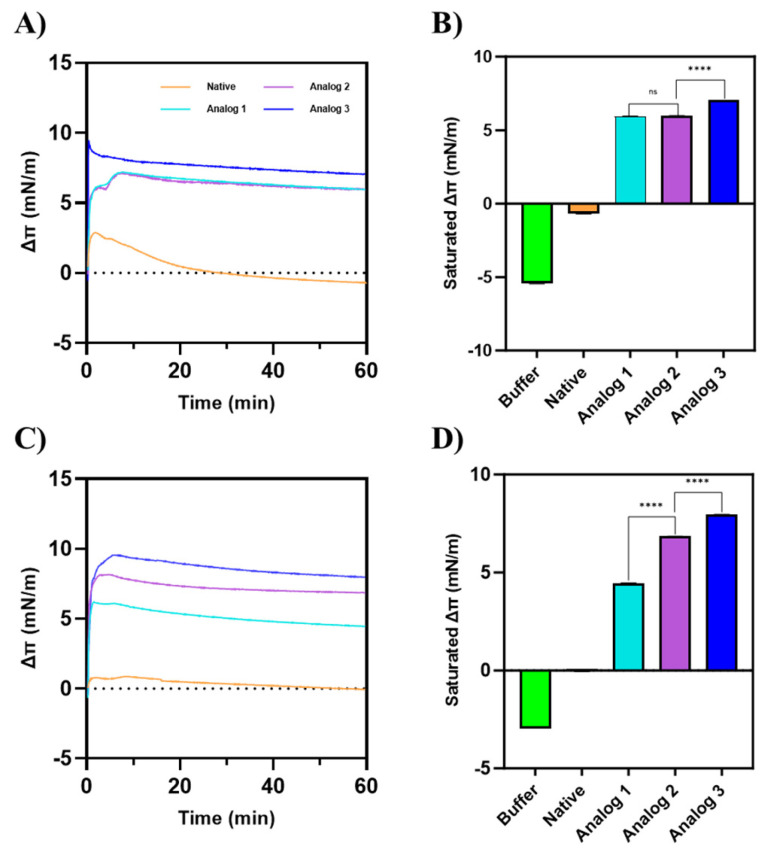
The change in the surface pressure (Δπ) of membranes by peptides on (**A**,**B**) a Gram-positive membrane model consisting of POPG:POPE with a 7:3 ratio and (**C**,**D**) a Gram-negative membrane model consisting of POPG:POPE with a 3:7 ratio. Analog-3 exhibited the highest surface activity on both membrane models. On a Gram-positive membrane model, Analog-2 showed no significant difference in surface activity compared to Analog-1. However, on the Gram-negative membrane model, Analog-2 demonstrated a higher Δ*π* than Analog-1. These findings indicate that the surface activity of each peptide is influenced by the composition of the membrane. Statistical comparisons between peptide groups were performed using a Mann–Whitney U test, a non-parametric, two-tailed test, with a significance level set at *p* < 0.05.

The hypothesis is that the antibacterial activities of native and analog peptides are due to their binding properties to bacterial membranes to disrupt membrane stability that kill bacteria. In this study, this hypothesis was tested by evaluating the membrane binding property of each peptide in two models of bacterial membranes. The first model was Gram-positive membranes that consist of POPG:POPE with a ratio of 7:3. The second model was Gram-negative membranes with a 3:7 ratio of POPE:POPG molecules. These model membranes were used because phosphatidylethanolamine (PE) and phosphoglycerol (PG) are the most abundant phospholipids present on the bacterial cell surface [37]. We proposed that the peptide can cross the outer membrane, followed by its interaction with the inner membrane bacteria to cause membrane disruption [38,39]. Insertion of peptide into the lipid membrane can show an increase in the surface pressure at the buffer–air interface (Figure 7).

For a model of Gram-positive membrane (Figure 7A,B), Analog-3 produced the highest change in surface pressure (Δ*π*), compared to Analog-1 and -2. Analog-1 and -2 exhibited similar Δ*π* values, which reached saturation around 6 mN/m. The native peptide did not show a meaningful change in the surface pressure before or after peptide injection, indicating that it interacted very weakly with membranes. In the Gram-negative membrane model (Figure 7C,D), the Δ*π* of Analog-3 was the largest, followed by Analog-2 and Analog-1. Unlike in the Gram-positive model, Analog-2 had a higher Δ*π* than Analog-1. The native peptide has a very small effect on the membrane surface pressure.

### 3.6. Antibacterial Activity of Peptides

To correlate helical stability and membrane binding properties with the antibacterial activity, the activity of each peptide to inhibit bacterial growth was evaluated in Gram-positive *S. aureus* and Gram-negative *E. coli*. In *S. aureus* bacteria, Analog-2 and -3 exhibited MIC values of 3.91 µg/mL for both (Table 4). For Analog-1, the MIC value is 125 µg/mL. The native peptide showed no antibacterial activity, even at 250 µg/mL. Moreover, in *E. coli*, Analog-2 and -3 have MIC values of 62.5 µg/mL for both, while Analog-1 again has a weaker activity with the MIC value of 125 µg/mL (Table 4). Overall, the Analog-2 and -3 have the best activity in both *S. aureus* and *E. coli* bacteria, and they have better activity in *S. aureus* compared to *E. coli*.

The results suggest that the helical stability and membrane binding properties of the native peptide and its analogs correlated with their antibacterial activities in both *S. aureus* and *E. coli*. The antibacterial activities of Analog-2 and -3 were comparable despite some differences in their helical content and membrane binding properties. The noticeable higher activity of Analog-2 and -3 in *S. aureus* compared to *E. coli* cannot be explained solely as a factor for their strong helical stability and membrane binding properties. Thus, other factors such as the overall sequence, charge distribution, and interactions with bacterial cell components can also play important roles in disrupting the bacterial growth. Capping the N- and C-terminus in Analog-2 and -3 as well as increasing amphipathicity have been shown to increase peptide stability, membrane binding properties, and antibacterial activity.

## 4. Discussion

**Figure 8:**
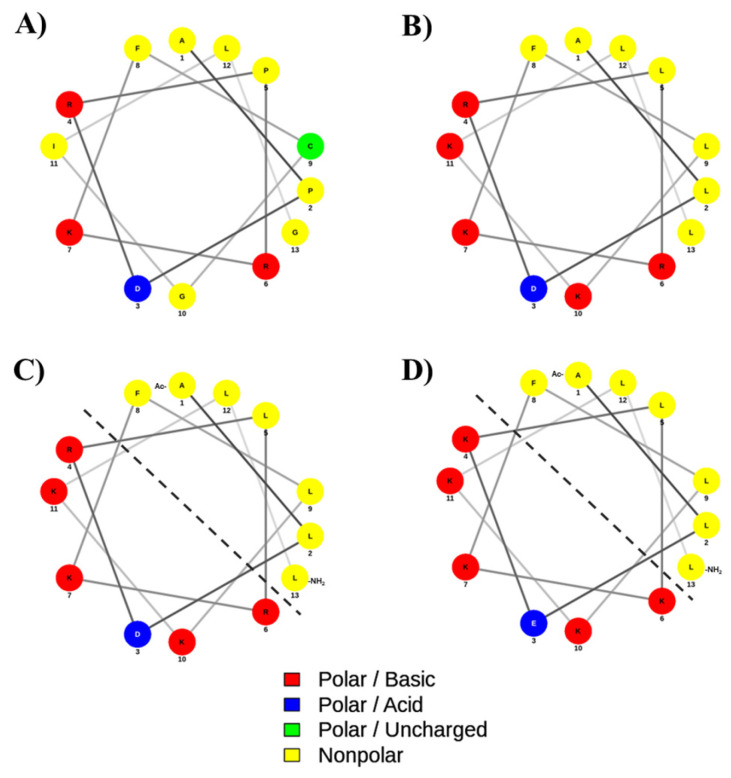
The helical wheel and net projection of (**A**) native, (**B**) Analog-1, (**C**) Analog-2, and (**D**) Analog-3. The dashed line separates the hydrophobic and hydrophilic surfaces of the amphipathic helix structure. The polar basic residues with a positive charge and polar acidic residues with a negative charge are indicated as red and blue colors, respectively. The non-polar and polar uncharged residues are marked as yellow and green colors, respectively. The helical diagram was generated using NetWheels: Peptides Helical Wheel and Net projections maker (http://www.lbqp.unb.br/NetWheels/, accessed on 7 August 2025).

In this study, we designed derivatives of Wuchuanin-A1 as antimicrobial peptides (AMPs) to potentially overcome bacterial resistance. One potential strategy is to design AMPs that bind and disrupt bacterial membranes to create bacterial membrane leakiness, and this method has been shown to minimize bacterial resistance [38,39,40]. Effectiveness of AMR to bind membranes can be accomplished by forming stable and amphipathic helix peptides from the native peptide, Wuchuanin-A1. It has been suggested by Tan et al. (2014) that simply substituting amino acids was not sufficient to improve peptide helical structure [41]. Therefore, strategically forming amphipathic structure as well as capping both N- and C-termini should be considered [41]. The formation of amphipathic helix has been shown to promote a clear separation between the hydrophilic and hydrophobic sides of the peptide that facilitates the alignment of hydrophobic faces between two or more molecules to form dimer or oligomers to stabilize the α-helical structure (Figure 8) [42,43]. Therefore, peptide analogs were designed to stabilize the α-helical structure.

The analog design was initiated by mutating helix breaker residues in the native peptide to Analog-1 by mutating Pro2-to-Leu2, Pro5-to-Leu5, Gly10-to-Lys10, and Gly13-to-Leu13 residues (Figure 8A,B). Additional mutations were carried out by changing Cys9-to-Leu9 and Ile11-to-Lys11 to improve the amphipathicity of the helix in Analog-1. Experimentally, Analog-1 has a higher α-helix content (*f_H_*) compared to the non-helical native peptide (Figure 1A,B). Next, the open N- and C-termini in Analog-1 were capped with acetyl and amide groups, respectively, to produce Analog-2 (Figure 8B,C). Capping of the N- and C-termini has been shown to improve the enzymatic stability in the biological system because it prevents the peptide degradation by exopeptidases such as amino- and carboxy-peptidases. The result showed that this capping enhanced maximum fraction of helix (*f_H_*) of Analog-2 compared to the Analog-1 (Figure 1E and Figure 2E). The *f_H_* values of Analog-2 were higher than Analog-1 at different concentrations of TFE (Figure 1E) and MeOH (Figure 2E). Analog-2 was further mutated to Analog-3 at several residues such as Asp3-to-Glu3, Arg4-to-Lys4, and Arg6-to-Lys6 (Figure 8C,D). Substitution of Asp-to-Glu and Arg-to-Lys residues increased the helical stability in Analog-3 because the Glu and Lys residues are strong helix formers [44,45]. Although both the Asp and Glu amino acids are negatively charged, the Glu amino acid is better than the Asp amino acid in stabilizing the α-helical structure because the Glu residue can form a salt bridge with the neighboring Arg or Lys residue within the peptide [46].

The experimental results showed further improvement in helical stability of Analog-3 compared to Analog-2. Analog-3 has higher *f_H_* values in various concentrations of TFE and MeOH compared to Analog-2, supporting the idea that Analog-3 has higher helical stability than Analog-2 (Figure 1E and Figure 2E). In this study, TFE was observed to be a better inducer of α-helical structure than MeOH, and this was notable in the weaker effect of MeOH (Figure 2E) than TFE (Figure 1E) in inducing the helicity of Analog-1. The ability of TFE to stabilize peptide helical structure was due to the generation of a membrane-like environment to drive the formation of intramolecular hydrogen bonding within the peptide [47]. Computational studies showed that TFE is significantly more effective than MeOH in promoting helicity because TFE as a solvent has lower interaction with the peptide compared to MeOH [48].

Modifications at both the N- and C-termini in these peptides increased their helical content and structural stability as well as increasing the dimerization or oligomerization properties (Table 2) compared to Analog-1. The formation of peptide oligomers upon binding to membranes can create pores to make bacterial membranes leaky (Figure 7) [49]. However, oligomerization alone may not fully explain their antibacterial action. For example, the monomer of Aurein 1.2 can form membrane-disrupting pores; in contrast, the dimer can form stable channels in the membrane, indicating that dimerization can alter the mechanism of action [49]. Similarly, our peptides may undergo structural changes upon oligomerization that impact their interaction with bacterial membranes. Although the N-and C-termini capping modification does not alter the overall peptide charge, they significantly impact α-helical stability of Analog-2 and -3. Previous studies have shown that these modifications can promote the α-helical content in peptide to enhance its functional properties [50] as well as helping to ‘lock’ the helical structure in place by preventing the unraveling of both ends of the peptide [51,52].

To confirm the helical stability of Analog-2 and -3, the solution conformations of these peptides were determined using NMR and MD simulations. The results showed that both Analog-2 and -3 have a continuous helical structure from N- to C-termini, as reflected by the continuous NH_(i)_-NH_(i+1)_ (Figure 5D and Figure 6D) and Hα_(i)_-NH_(i+1)_ (Figure 5C and 6C) interactions in the NOE spectra. The generated structures from NMR-restrained MD simulations correlated well with the interproton distances used during MD simulations. The resulting low-energy structures have the Phi/Psi angles (Table 3) in the helical region of the Ramachandran plot (Figure 5G and Figure 6G). Both Analog-2 and -3 were more stable in different pHs compared to Analog-1 and the native peptide (Figure 4). The helical conformations for both Analog-2 and -3 were stable in different time points in MD simulations (Supplementary Figure S3). The increased stability is essential for resisting environmental stress in bacterial surroundings. The α-helical stability against temperature changes was due to the formation of intramolecular hydrogen bonds as well as the salt bridge formation within the peptide (Figure 3) [53]. Additionally, the stable hydrogen-bonding network in α-helical structure allows peptide to resist disruptions in structure caused by alteration in protonation state as pH fluctuates (Figure 4). Thus, the modifications to these peptides not only enhance helicity but also improve their overall stability under various conditions.

The membrane binding properties of these peptides can be correlated to their amphipathicity of the peptides (Figure 7). The amphipathicity of the peptides was reflected in their hydrophobic moment (µH) values, with Analog-1, -2, and -3 showing high µH values, consistent with their stronger membrane binding and antibacterial activity (Table 1). Higher µH values indicate a stronger segregation of hydrophobic and hydrophilic residues, which promotes more effective alignment with bacterial membranes. The hydrophobic moments were calculated using HeliQuest based only on the amino acid sequence; therefore, terminal capping groups were not included in the calculation [54]. As a result, Analog-1 and -2 exhibited the same highest µH value (0.798), while Analog-3 displayed a slightly lower value (0.789). All three analogs, however, had markedly higher µH compared to the native peptide (0.498). Furthermore, Takechi-Haraya et al. (2022) demonstrated with A2-17 peptide isomers that when the hydrophobic moment becomes too high, peptides may preferentially associate with the plasma membrane rather than penetrate efficiently [55]. Therefore, the slightly lower µH of Analog-3 may represent a more optimal balance for effective membrane insertion.

Both Analog-2 and -3 have the highest helical amphipathic properties with the best antibacterial activities against *S. aureus*. It is interesting to observe that the antibacterial activities of both Analog-2 and -3 were higher against Gram-positive *S. aureus* compared to Gram-negative *E. coli*. In addition, Analog-2 and -3 still have better activity against *E. coli* compared to Analog-1 and the native peptide. Compared with other peptides, including Wuchuanin-A1 and related types such as Wuchuanin-C1 and D1, our analog peptides demonstrated markedly better antibacterial activity, as evidenced by their lower MIC values. According to Yang et al., (2012), Wuchuanin-A1, C1, and D1 exhibited MIC values >200 µg/mL against *E. coli* and *S. aureus* [13]. On the other hand, in our study, using the same bacterial strains, Analog-1 had 125 µg/mL MIC values for both *E. coli* and *S. aureus*, whereas Analog-2 and -3 were even more active with MIC values of 62.5 µg/mL for *E. coli* and as low as 3.91 µg/mL for *S. aureus*. Moreover, our analogs showed good antibacterial potential compared with our positive control, chloramphenicol (MIC values: 3.91 µg/mL for *E. coli* and 7.81 µg/mL for *S. aureus*). The activity is also similar to other well-studied frog-skin peptides such as brevinin, nigrocin, odorranain, and esculentin, and demonstrates the potent activity of our peptide analogs [13].

These observations correlate with the higher binding properties of Analog-2 and -3 to both models of Gram-positive and Gram-negative membranes compared to Analog-1 and the native peptide (Figure 7). We proposed that the antibacterial activities of these peptides against both *E. coli* and *S. aureus* bacteria were due to their ability to disrupt bacterial cell membranes and cause leakage in the bacterial cell wall. In this case, the peptide can be inserted or incorporated into the cell membranes to change the membrane structure and possibly to create pores due to membrane leakiness due to the formation of organized oligomers in the membranes [56]. Overall, the effects on amphipathicity, helical stability, peptide sequence, and oligomerization on bacterial membrane disruptions cannot be easily separated. Thus, there is a need to further investigate the contribution of different peptide properties to its antibacterial activity.

The stronger activity of Analog-2 and -3 against Gram-positive bacteria *S. aureus* compared to Gram-negative *E. coli* can be attributed to differences in cell membrane structures. Gram-negative bacteria possess an outer membrane with lipopolysaccharides (LPS) and porins, forming a selective barrier to antimicrobial agents. This membrane not only restricts peptide entry but also enhances resistance through efflux pumps to actively expel antimicrobial agents. In contrast, Gram-positive bacteria lack this outer membrane but have a thick peptidoglycan layer, which is generally more permeable to peptides [57,58]. The antibacterial activity of Analog-2 was similar to Analog-3 although Analog-3 has higher helical stability than Analog-3. In this case, a factor to consider is that the antimicrobial activity has reached a maximum threshold further beyond the potential increase in α-helical content of the peptide. In other words, because both Analog-2 and -3 have adopted a stable helical conformation, they may already be optimized for membrane interaction [59].

The binding properties of all the peptides to the two model membranes with varying surface charges showed significant differences. Overall, regardless of the surface charge in the membranes, in both model membranes, the native peptide showed no surface activity, while the analogs showed a positive change in surface pressure, suggesting that the helicity increases the surface activity and their ability to interact with the membranes. In the Gram-positive model, the membranes have a higher POPG than POPE content; thus, the membranes are more negatively charged compared to the Gram-negative model membranes. In the Gram-positive membrane, Analog-3 has the highest membrane binding properties or the highest change in surface pressure (Δ*π*), followed by Analog-2 and -1 (Figure 7A,B); this can be attributed to the highest helical stability of Analog-3 in TFE and MeOH (Figure 1E and Figure 2E). The lack of difference in Δ*π* or membrane binding properties between Analog-1 and -2 suggests that the capping of the N- and C-termini in Analog-2 has little effect on their interaction with the Gram-positive membrane. In contrast, a more zwitterionic Gram-negative membrane with a higher POPE than POPG content, the capping of N- and C-termini in Analog-2 enhanced the peptide interaction with the membranes compared to uncapped Analog-1. The most stable Analog-3 has the best binding property to Gram-negative membranes compared to other peptides. MD simulations study showed how the stability of an α-helix peptide may vary depending on the Gram-positive and -negative environments, and its antimicrobial activity correlates with its α-helical stability upon interaction with bacterial membrane [60].

## 5. Conclusions

This study showed that there was a correlation between α-helical stability and amphipathic structure with membrane binding and antibacterial properties of Wuchuanin-A1 and its analogs (Analog-1, -2, and -3). Analog-3 has the highest α-helical stability, as determined by CD, NMR, and MD simulations. The helical content of Analog-2 is slightly lower than Analog-3 but better than Analog-1 and the native structure. Both Analog-2 and -3 effectively inhibit the growth of Gram-positive *S. aureus* compared to Analog-1 and the native peptide (Wuchuanin-A1). Although Analog-2 and -3 are less effective for Gram-negative *E. coli*, they are more effective than Analog-1 and the parent Wuchuanin-A1. These analogs were more potent than the previously reported Wuchuanin peptides, with low antibacterial effect. This increase in activity is probably explained by the fact that they can interact and interfere with bacterial membranes. Future studies could further investigate their membrane-disruptive mechanisms and their antibacterial activity on resistant strains.

## Figures and Tables

**Table 1:** Sequences and physicochemical properties of peptides.

Peptide	Sequence	MW (Da)	Net Charge (pH = 7)	pI	Hydrophobicity (H%)	Hydrophobic Moment (µH)
Native	APDRPRKFCGILG	1429.68	2	10.17	38.46	0.498
Analog-1	ALDRLRKFLKKLL	1614.01	4	11.5	53.85	0.798
Analog-2	Ac-ALDRLRKFLKKLL-NH_2_	1655.05	4	12.18	53.85	0.798
Analog-3	Ac-ALEKLKKFLKKLL-NH_2_	1613.06	4	11.27	53.85	0.789

**Table 2:** Dissociation constant value of peptides.

Peptide	*K_d_* (M)	[*θ*] Monomer (deg cm^2^ dmol^−1^)	[*θ*] Complex (deg cm^2^ dmol^−1^)
Analog-1	2 × 10^−4^	−92,000	−106,600
Analog-2	1 × 10^−4^	−360,000	−130,000
Analog-3	2.5 × 10^−5^	−450,000	−140,000

**Table 3:** ^3^*J_NH-HCα_* from NMR used to calculated Phi dihedral angles for MD simulations and the observed Phi dihedral angles from MD simulations of Analog-2 and Analog-3.

Analog-2				Analog-3			
Residue	^3^*J_NH-HCα_* (Hz)	NMR Calculated Phi	MD Observed Phi	Residue	^3^*J_NH-HCα_* (Hz)	NMR Calculated Phi	MD Observed Phi
Ala1	4.8	−64.93	360	Ala1	4.2	−60	360
Leu2	6.0	−74.30	−60.16	Leu2	6.6	−78.98	−78.45
Asp3	6.0	−74.30	−33.70	Glu3	5.4	−69.66	−60.77
Arg4	5.4	−69.66	−62.09	Lys4	6.0	−74.30	−57.39
Leu5	6.6	−78.98	−68.14	Leu5	6.6	−78.98	−73.82
Arg6	6.0	−74.30	−68.05	Lys6	6.0	−74.30	−70.66
Lys7	6.0	−74.30	−60.57	Lys7	6.0	−74.30	−67.05
Phe8	6.6	−78.98	−64.47	Phe8	6.0	−74.30	−56.36
Leu9	6.0	−74.30	−64.41	Leu9	6.6	−78.98	−67.29
Lys10	6.0	−74.30	−53.87	Lys10	6.0	−74.30	−53.17
Lys11	7.2	−83.82	−61.54	Lys11	6.0	−74.30	−72.42
Leu12	6.6	−78.98	−63.53	Leu12	6.6	−78.98	−95.22
Leu13	6.6	−78.98	−86.19	Leu13	6.6	−78.98	−88.05

**Table 4:** Antibacterial activities (MIC) of AMPs against *S. aureus* and *E. coli.*

Compound	MIC (µg/mL)	
	*S. aureus* (ATCC 25923)	*E. coli* (ATCC 25922)
Native	NA	NA
Analog-1	125	125
Analog-2	3.91	62.50
Analog-3	3.91	62.50
Chloramphenicol	7.81	3.91

NA: Not active in the tested concentration range (0.49–250 µg/mL).

## Data Availability

We have provided additional data in the Supplementary Materials.

## Supplementary Materials

The following supporting information can be downloaded at: https://www.mdpi.com/article/10.3390/life15101568/s1: Figure S1: HPLC traces and Mass Spectrometry data for (**A**) native peptide, (**B**) Analog-1, (**C**) Analog-2, and (**D**) Analog-3; Figure S2: Concentration-dependent changes in the CD signal at 222 nm for Analog-1, -2, and -3; Figure S3: Conformational stability of the helical conformation of Analog-2 and -3 at different time points during MD Simulations; Table S1: Proton chemical shifts in Analog-2 determined by COSY and TOCSY; Table S2: Interproton distance calculation of Analog-2; Table S3: Dihedral angle after MD simulations of Analog-2; Table S4: Proton chemical shift in Analog-3 determined by COSY and TOCSY; Table S5: Interproton distance calculation of Analog-3; Table S6: Dihedral angle after MD simulations of Analog-3.

## Abbreviations

1D-NMR	One-dimensional ^1^H NMR
2D-NMR	Two-dimensional nuclear magnetic resonance
AMP	Antimicrobial peptide
ARIA	Ambiguous Restraints for Iterative Assignment
CD	Circular dichroism
CLSI	Clinical lab standards institute
COSY	Correlation Spectroscopy
*E. coli*	*Escherichia coli*
*f_H_*	Mean helicity value
TFE	Trifluoroethanol
MeOD	Deuterated methanol
MeOH	Methanol
MD	Molecular dynamics
MRE	Mean residue molar ellipticity
NOESY	Nuclear Overhauser Effect Spectroscopy
PME	Particle Mesh Ewald
RMSD	Root-mean square deviation
*S. aureus*	*Staphylococcus aureus*
TOCSY	Total Correlation Spectroscopy
