# Supplementary material for: Design, Structural Stability, Membrane Binding, and Antibacterial Activity of Novel Antimicrobial Peptides Derived from Wuchuanin-A1
Source: Life (Basel). 2025 Oct 8;15(10):1568. doi: 10.3390/life15101568 (PMC12565435; doi:10.3390/life15101568)
Supplement: Supplementary file 1 [file life-15-01568-s001.zip › life-3837929-supplementary.pdf]

A)

HPLC Trace of Native Peptide

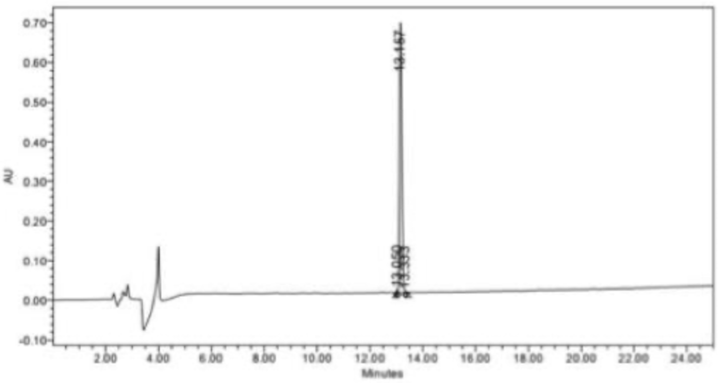

	RT	Area	% Area	Height
1	13.050	14658	0.37	7068
2	13.157	3984826	99.42	680404
3	13.333	8409	0.21	2756

MS Spectrum of Native Peptide

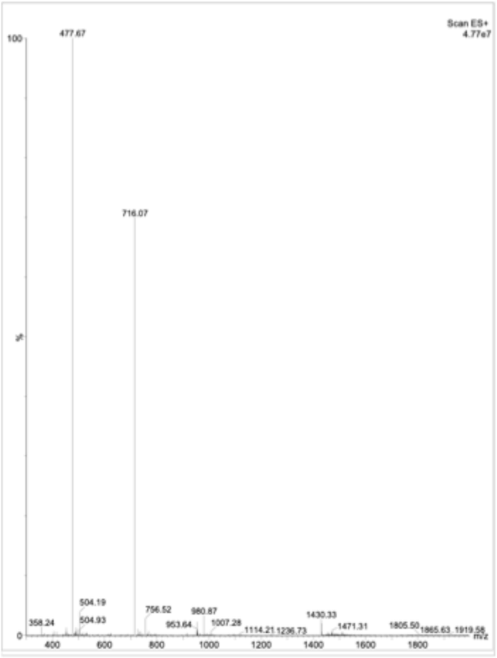

B)

HPLC Trace of Analog-1

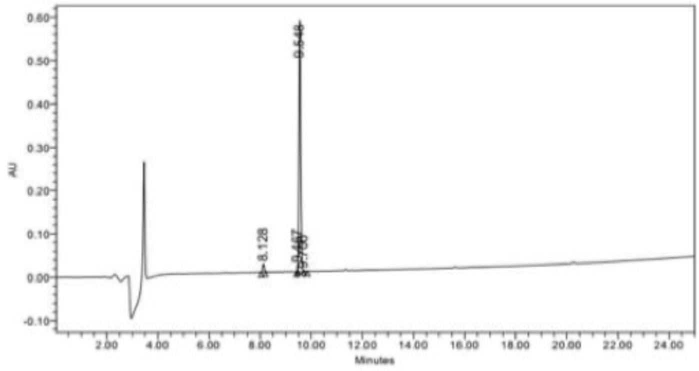

	RT	Area	% Area	Height
1	8.128	83719	2.82	18828
2	9.467	10780	0.36	16048
3	9.548	2861030	96.52	571796
4	9.700	8758	0.30	1804

MS Spectrum of Analog-1

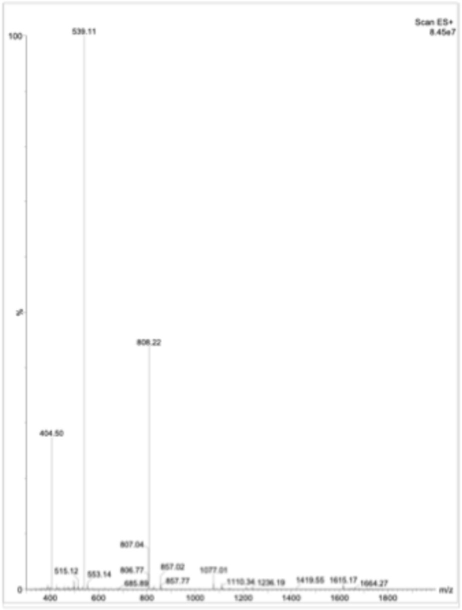

C)

HPLC Trace of Analog-2

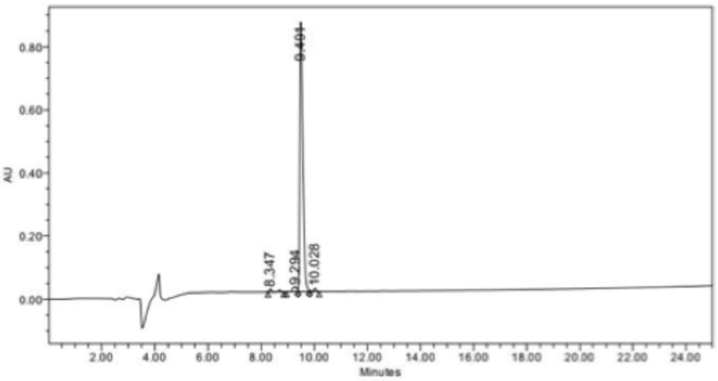

	RT	Area	% Area	Height
1	8.347	84345	1.12	9244
2	9.294	164021	2.17	15822
3	9.491	7228924	95.75	863622
4	10.028	72285	0.96	10439

MS Spectrum of Analog-2

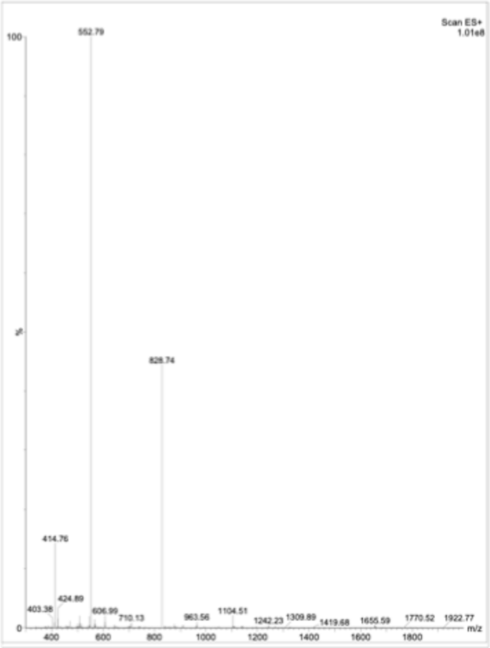

D)

HPLC Trace of Analog-3

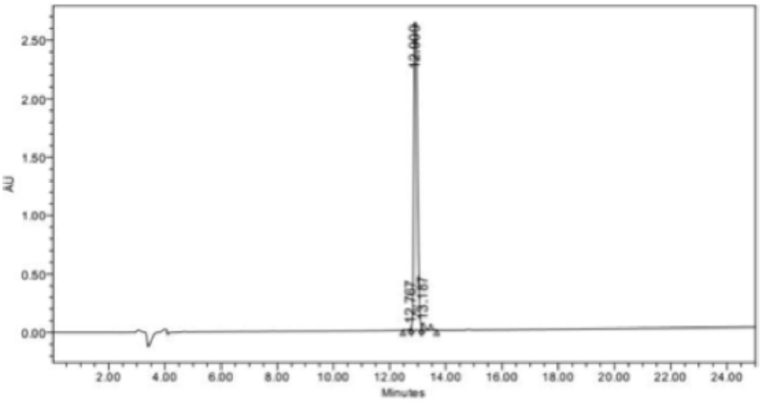

	RT	Area	% Area	Height
1	12.767	71110	0.31	24691
2	12.909	21989633	96.67	2666787
3	13.187	686774	3.02	58945

MS Spectrum of Analog-3

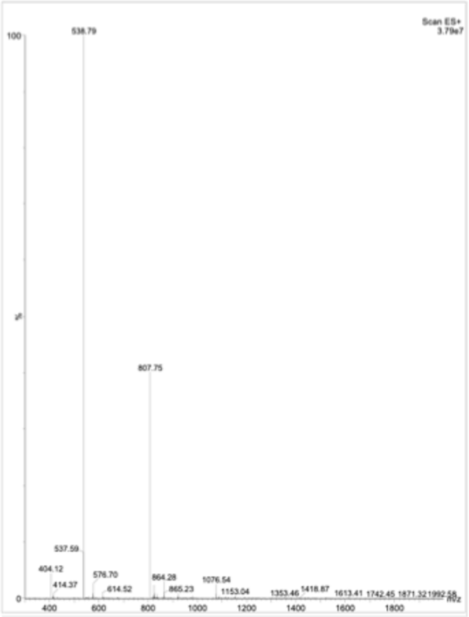

Figure S1. HPLC traces and Mass Spectrometry data for **(A)** Native Peptide, **(B)** Analog-1, **(C)** Analog-2, and **(D)** Analog-3.

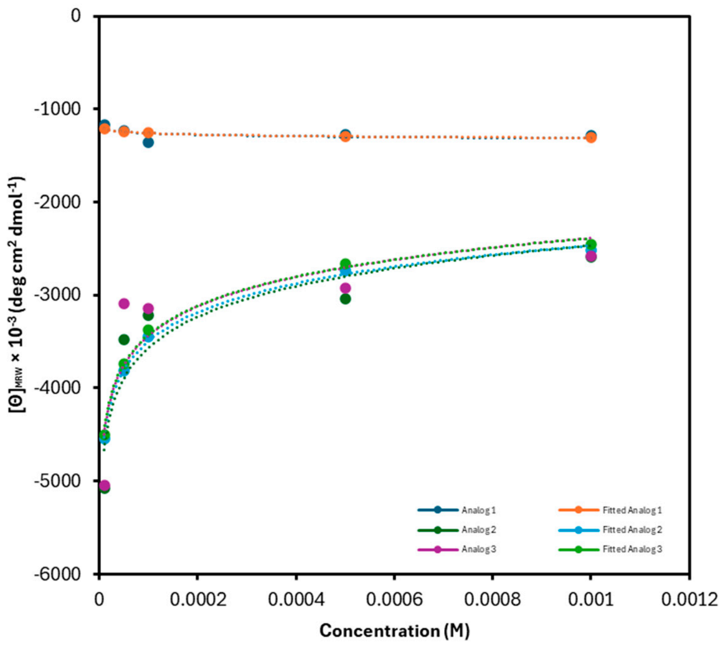

Figure S2. Concentration-dependent changes in the CD signal at 222 nm for Analog-1, -2, and -3.

Analog-2:

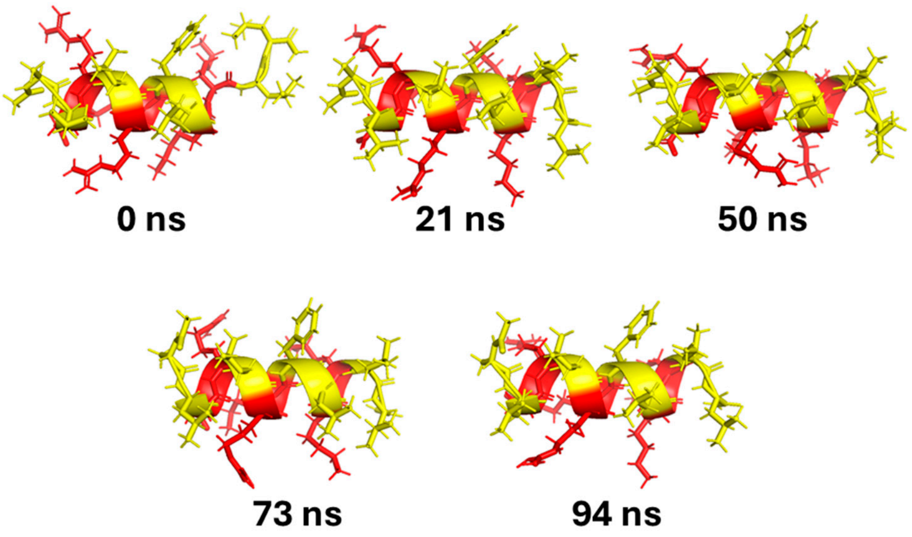

Analog-3:

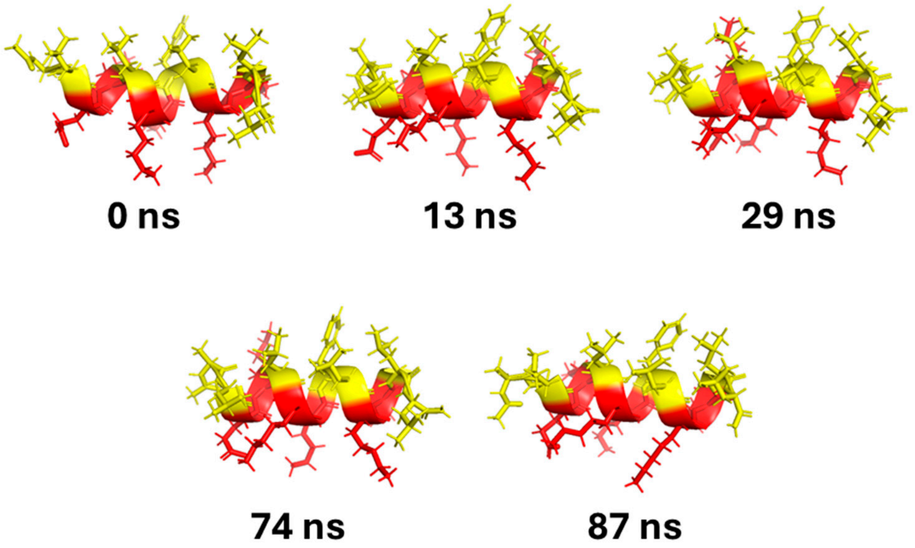

Figure S3. Conformational stability of the helical conformation of Analog-2 and -3 at different time points during MD Simulations.

Table S1. Proton chemical shifts of Analog-2 determined by COSY and TOCSY.

Residue	NH	Hα	Hβ	Hγ	CH_2	CH_3
Acetyl	-	-	-	-	-	0.91<br>1.66
A1	8.39	4.15	-	-	-	1.4
L2	8.38	4.24	1.66	-	-	0.93
D3	8.13	4.5	2.78	-	-	-
R4	8.08	4.12	1.88<br>1.71	-	1.61<br>3.22	-
L5	7.98	4.29	1.74<br>1.58	-	-	0.90
R6	8.12	4.07	1.86<br>1.75	-	1.61<br>3.21	-
K7	8.01	4.11	1.74<br>1.43	-	1.31<br>2.95	-
F8	8.05	4.50	3.25<br>3.12	-	-	-
L9	8.19	4.15	1.75<br>1.53	-	-	0.93
K10	8.00	4.11	1.74<br>1.43	-	1.31<br>2.95	-
K11	7.93	4.18	1.81<br>1.66	-	1.44	-
L12	8.04	4.22	1.57	-	-	0.86
L13	7.99	4.29	1.74<br>1.58	-	-	0.90
NH_2 (Amide)	7.08<br>7.32	-	-	-	-	-

Table S2. Interproton distance calculation of Analog-2.

**Cross peaks**	**Hα**	**NH**	**NH**	**HCβ**	**Intensity**	**Distance**
$H\alpha_{(i)}$-$NH_{(i+1)}$	Ala1	Leu2			60.55	2.89
	Leu2	Asp3			307.08	2.20
	Asp3	Arg4			159.96	2.45
	Leu5	Arg6			307.08	2.20
	Lys7	Phe8			325.19	2.18
	Phe8	Leu9			222.85	2.32
	Lys10	Lys11			443.84	2.07
$NH_{(i)}$-$NH_{(i+1)}$		Leu2	Asp3		153.92	2.47
		Asp3	Arg4		54.51	2.94
		Arg4	Leu5		184.31	2.40
		Leu5	Arg6		497.5	2.03
		Arg6	Lys7		517.54	2.02
		Phe8	Leu9		101.34	2.65
		Leu9	Lys10		300.7	2.21
		Lys10	Lys11		228.07	2.31
		Lys11	Leu12		375.07	2.13
$NH_{(i)}$-$NH_{(i+2)}$		Leu2	Arg4		21.17	3.44
		Arg6	Phe8		18.41	3.52
		Leu9	Lys11		31.32	3.22
$H\alpha_{(i)}$-$NH_{(i+3)}$	Arg6		Leu9		59.19	2.89
	Phe8		Lys11		19.48	3.49
$H\alpha_{(i)}$-$NH_{(i+4)}$	Asp3		Lys7		55.83	2.93
	Leu5		Leu9		17.73	3.54
$H\alpha_{(i)}$-$H\beta_{(i+3)}$	Leu2			Leu5	64.88	2.85
	Asp3			Arg6	141.57	2.51
					84.33	2.73
	Leu5			Phe8	74.6	2.79
					42.30	3.07
	Phe8			Lys11	27	4.14
					20.6	4.55

Table S3. Dihedral angle after MD simulations of Analog-2.

Residue	Structure	Phi (calculation)	Phi (simulation)	Psi (simulation)
A1	Coil	-64.93	360	142.63
L2	α-Helix	-74.30	-60.16	-45.41
D3	α-Helix	-74.30	-33.70	-60.22
R4	α-Helix	-69.66	-62.09	-45.14
L5	α-Helix	-78.98	-68.14	-41.02
R6	α-Helix	-74.30	-68.05	-38.84
K7	α-Helix	-74.30	-60.57	-37.01
F8	α-Helix	-78.98	-64.47	-39.62
L9	α-Helix	-74.30	-64.41	-37.73
K10	α-Helix	-74.30	-53.87	-51.07
K11	α-Helix	-83.82	-61.54	-41.67
L12	α-Helix	-78.98	-63.53	-43.89
L13	Coil	-78.98	-86.19	

Table S4. Proton chemical shift of Analog-3 determined by COSY and TOCSY.

Residue	NH	Hα	Hβ	Hγ	CH_2	CH_3
Acetyl	-	-	-	-	-	0.91<br>1.70
A1	8.4	4.15	-	-	-	1.48
L2	8.37	4.23	1.66	-	-	0.89
E3	8.12	4.14	2.06	2.42	-	-
K4	7.98	4. 11	1.85<br>1.67	-	1.41<br>2.96	-
L5	7.89	4.3	1.7	-	-	0.91
K6	8.12	4.07	1.80<br>1.66	-	1.37<br>2.92	-
K7	7.97	4.12	1.74<br>1.62	-	1.30<br>2.91	-
F8	8.03	4.52	3.23<br>3.09	-	-	-
L9	8.17	4.18	1.68<br>1.52	-	-	0.89
K10	8.02	4.16	1.82<br>1.69	-	1.46<br>2.97	-
K11	7.99	4.2	1.85<br>1.67	-	1.41<br>2.96	-
L12	8.08	4.25	1.57	-	-	0.85
L13	8.02	4.28	1.68<br>1.59	-	-	-
NH_2 (Amide)	7.08<br>7.36	-	-	-	-	-

Table S5. Interproton distance calculation of Analog-3.

**Cross peaks**	**Hα**	**NH**	**NH**	**HCβ**	**Intensity**	**Distance**
$H\alpha_{(i)}$-$NH_{(i+1)}$	Ala1	Leu2			79.76	2.76
	Leu2	Glu3			123.03	2.57
	Lys4	Leu5			174	2.42
	Leu5	Lys6			226.42	2.32
	Lys6	Lys7			446.91	2.07
	Lys7	Phe8			458.18	2.06
	Phe8	Leu9			260.73	2.27
	Lys11	Leu12			348.30	2.16
$NH_{(i)}$-$NH_{(i+1)}$		Ala1	Leu2		36.59	3.14
		Leu2	Glu3		86.07	2.72
		Glu3	Lys4		264.1	2.26
		Lys4	Leu5		210.02	2.35
		Leu5	Lys6		152.61	2.48
		Lys6	Lys7		264.1	2.26
		Lys7	Phe8		407.49	2.10
		Phe8	Leu9		384.92	2.12
		Leu9	Lys10		352.46	2.15
		Lys11	Leu12		209.59	2.35
		Leu12	Leu13		648.49	1.95
$NH_{(i)}$-$NH_{(i+2)}$		Ala1	Glu3		2.45	4.93
		Leu2	Lys4		14.72	3.66
		Leu5	Lys7		18.32	3.53
$H\alpha_{(i)}$-$NH_{(i+3)}$	Leu2		Leu5		14.94	3.65
	Arg6		Leu9		88.03	2.71
	Phe8		Lys11		32.95	3.20
$H\alpha_{(i)}$-$NH_{(i+4)}$	Leu5		Leu9		17.53	3.55
	Phe8		Leu12		7.61	4.08
	Leu9		Leu13		40.49	3.09
	Leu5			Phe8	40.83	3.09
					19.79	3.48
	Lys6			Leu9	62.5	2.87
	Phe8			Lys11	8.72	3.99
					12.91	3.74

Table S6. Dihedral angle after MD simulations of Analog-3.

Residue	Structure	Phi (calculation)	Phi (simulation)	Psi (simulation)
A1	α-Helix	-60	360	-42.46
L2	α-Helix	-78.98	-78.45	-31.85
E3	α-Helix	-69.66	-60.77	-40.22
K4	α-Helix	-74.30	-57.39	-45.69
L5	α-Helix	-78.98	-73.82	-37.02
K6	α-Helix	-101.72	-70.66	-37.08
K7	α-Helix	-74.30	-67.05	-36.99
F8	α-Helix	-78.98	-56.36	-55.37
L9	α-Helix	-78.98	-67.29	-36.93
K10	α-Helix	-74.30	-53.17	-40.57
K11	α-Helix	-74.30	-72.42	-17.75
L12	α-Helix	-78.98	-95.22	-14.13
L13	Coil	-74.30	-88.05	
